# Relationship between dry eye and expressions of CXCR3 and CCR5 after ocular acid burn

**DOI:** 10.1186/s12886-022-02678-3

**Published:** 2022-12-15

**Authors:** Bo Jiang, Qianqian Hu, Tao Li, Man She, Chunxia Li, Xiaodong Zhou

**Affiliations:** 1 Shanghai University of Traditional Chinese Medicine, Shanghai, China; 2 Department of Ophthalmology, Jinshan Hospital Affiliated to Fudan University, Shanghai, China; 3 Department of Ophthalmology, Huadong Hospital Affiliated to Fudan University, Shanghai, China; 4 Department of Ophthalmology, Shanghai TCM-Integrated Hospital, Shanghai University of TCM, Shanghai, China

**Keywords:** Burn, Acid, Ocular, CXCR3, CCR5

## Abstract

**Objective:**

To investigate the manifestation of dry eye and its relationship with CXCR3 and CCR5 expression in patients with ocular acid burns.

**Methods:**

This is a case–control study. A total of 27 eyes of 22 cases ocular with acid burns of I-V degrees from Jan.2020 to Feb.2021 in Jinshan Hospital of Fudan University were selected as observation group, and 8 eyes of 8 cases of normal people were selected as control group. The follow-up period was 3 months. The visual acuity, intraocular pressure (IOP), corneal fluorescein staining scores (CFS), breakup time of tear film (BUT), Schirmer I test, corneal thickness and tear meniscus height (TMH) were observed at 1 day, 1 and 3 months after injury. The protein expressions of CXCR3 and CCR5 were examined by ELISA and compared among groups at each time point.

**Results:**

BUT and Schirmer I tests value in the observation group were lower than those in the control group 3 months after injury (BUT: Group I ~ IV *p* = 0.0266, *p* = 0.0222, *p* = 0.0003, *p* = 0.0059, respectively; Schirmer I test: Group I ~ IV *p* = 0.0027, *p* = 0.0033, *p* = 0.0016, *p* = 0.0032, respectively). CFS scores were higher than those in the control group at 1 day after injury (all *p* < 0.0001), but decreased gradually at 1 and 3 months after injury (Group I ~ IV *p* = 0.0042, *p* = 0.0096, *p* < 0.0001, *p* < 0.0001, respectively). The corneal thickness and TMH 1 day after injury were higher than those in the control group (corneal thickness: Group II ~ IV *p* = 0.0010, *p* < 0.0001, *p* < 0.0001, respectively; TMH: Group II ~ IV *p* = 0.0002, *p* < 0.0001, *p* < 0.0001, respectively), and also higher than those at 1 month and 3 months after injury (corneal thickness: Group II ~ IV *p* = 0.0010, *p* < 0.0001, *p* < 0.0001, respectively; TMH: Group II ~ IV *p *= 0.0345 and *p *= 0.0045, *p* = 0.0005 and *p* < 0.0001, *p* = 0.0114 and *p *= 0.0019, respectively). The expression levels of CXCR3 and CCR5 protein were significantly negatively correlated with BUT (all *p* < 0.0001), and CXCR3 and CCR5 were also significantly negatively correlated with Schirmer I test value (*p* < 0.0001, *p *= 0.0004, respectively).

**Conclusion:**

Ocular acid burns can cause dry eye, and the expression of CXCR3 and CCR5 protein in tears may be related to the occurrence of dry eye after ocular acid burn.

## Background

Ocular chemical burn, including acid burn and alkali burn, is one of the common eye injuries, accounting for 22% of ocular trauma [1], with acute onset and rapid progression, often leading to a variety of complications. And dry eye is one of the most common complications of acid burn, which may be related to the reduction of mucin production and the occurrence of immune inflammatory reaction caused by the keratoconjunctival epithelial damage after chemical eye injury [2]. Therefore, the diagnosis, preventive and treatment of dry eye after ocular acid burn have attracted much attention. According to Tear Film & Ocular Surface Society Dry Eye Workshop II (TFOS DEWS II), dry eye disease (DED) is a multi-factor eye disease correlation with the inflammation of ocular surface, characterized by insufficient tear production and imbalance of tear film homeostasis, accompanied by uncomfortable symptoms, in which tear film instability and hyperosmolarity, ocular surface inflammation and damage, and neurosensory abnormalities play etiological roles [3]. In the studies of various types of dry eye disease, furthermore, the increase of ocular surface inflammatory factors and chemokines has been found to induce immune inflammatory response, which may be an important co-pathogenesis of dry eye disease [4–6]. Among them, chemokine receptors CXCR3 (CXC chemokine receptor-3, CXCR3) and CCR5 (CC chemokine receptor-5, CCR5) act as surface markers of activated Th1 lymphocytes to mediate ocular surface inflammation [7]. Therefore, it may be of clinical significance to investigate the relationship between dry eye and expression of CXCR3 and CCR5 after ocular acid burn. In this study, the regularity of dry eye manifestations after acid burn and its relationship with CXCR3 and CCR5 were studied, providing clinical reference for the diagnosis and treatment of dry eye after acid burn.

## Materials and methods

### Patients

This study collected data from twenty-two patients with a primary diagnosis of ocular acid burn in the Department of Ophthalmology, Jinshan Hospital affiliated to Fudan University, China, from January 2020 to February 2021. All patients were diagnosed according to the International Classification of Diseases, Tenth Revision, Clinical Modification (ICD-10-CM). This study was approved by the Ethics Committee of Jinshan Hospital of Fudan University (IEC-2020-S03). All procedures conform to the principles of the Declaration of Helsinki. Written informed consent was obtained from all patients.

#### Inclusion criteria

(1) The patient was definitely diagnosed with acid ocular burn. (2) The patient had no history of dry eye, contact lens wearing, or eye surgery. (3) The patient didn’t have glaucoma, chronic dacryocystitis and eye diseases. (4) Patient was excluded from sjogren's syndrome, connective tissue disease, diabetes and other related diseases.

#### Clinical data

Twenty-two patients (27 eyes) with acid ocular burn treated in our hospital from January 2020 to February 2021 were selected as the observation group, including 18 males (22 eyes) and 4 females (5 eyes), ranging in age from 28 to 62 years, with an average age of 44.0 ± 9.6 years. The visit time was 30 min to 24 h after the injury, and all patients had their eyeballs irrigated with water at the scene. The degree of burn was in accordance with GBZ 54–2017 diagnostic criteria for occupational chemical eye burn [8]. The patients were divided into four groups: group I (grade I, *n* = 8 eyes), group II (grade II, *n* = 8 eyes), group III (grade III, *n* = 8 eyes), and group IV (grade IV and grade V, *n* = 3 eyes).

At the same time, 8 normal people without dry eyes (8 eyes) were selected as the control group, including 5 males and 3 females, aged 28–56 years, with an average age of 40.5 ± 9.0 years, and there was no significant difference in age from the observation group (*P* < 0.05).

### Methods

#### Treatment

The conjunctival sac of all injured patients was thoroughly rinsed with normal saline immediately after injury in the hospital, followed by topical antibiotic eye drops, eye ointment (levofloxacin eye drops, levofloxacin eye ointment) and eye drops that promote corneal repair (recombinant bovine basic fibroblast growth factor eye drops). Local glucocorticoid eye drops (tobramycin and dexamethasone eye drops) were administered within 1 week after injury, and amniotic membrane transplantation was performed in patients with grade III or higher burns.

#### Observation indicators

Visual acuity, intraocular pressure (IOP), corneal fluorescein staining scores (CFS), breakup time of tear film (BUT), Schirmer I tests, corneal thickness and TMH were performed in the control group and observation group at 1 day, 1 month and 3 months after injury, respectively. Meanwhile, the protein expression levels of CXCR3 and CCR5 were determined by Elisa.

- (1) Visual acuity and intraocular pressure (IOP): A standard logarithmic visual acuity chart and a NT-510 NCT (non-contact tonometer, Nidek, Japan) were used to measure patients' visual acuity and intraocular pressure, respectively.
- (2) Corneal fluorescein staining scores (CFS). The cornea was stained with 0.2% sodium fluorescein and negative staining indicated the integrity of the corneal epithelial cells. CFS used the 12-point method [9]: the cornea was divided into four quadrants, each quadrant can be scored 0–3 points according to the following criteria: 0, no spot dyeing; 1, 1–30 spots dyeing; 2, > 30 spots dyeing but not fused into tablets; 3, corneal spots dyed point fusion or ulcers.
- (3) Breakup time of tear film (BUT): 1% fluorescein sodium was dropped into the conjunctiva sac, and slit lamp observation was performed after several blinks. BUT was the time from eye opening after the last blink to the first black spot on the cornea. Average value was taken for three consecutive measurements, normal BUT was ≥ 10 s.
- (4) Schirmer I test: The Schirmer filter paper was placed in the conjunctiva sac of the subject under normal indoor light, and removed after 5 min to measure the wetting length, which was the value of Schirmer I test. The wetting length of the filter paper was ≥ 10 mm after 5 min in normal subjects.
- (5) Measurement of TMH and corneal thickness: Cirrus-HD 4000 OCT (optical coherent tomography, Zeiss, Germany) was adopted. The light source was near infrared light at 1310 nm. The scanning range was 10 mm in the transverse direction and 3 mm in the longitudinal direction, with resolutions of 19 um and 4 um respectively. Adopt the single-line scanning mode of the internal fixation device to adjust the fixation angle so that the optical axis is consistent with the optical axis. The high resolution mode was selected, and the scan line was adjusted to the tear meniscus below the vertical cornea center. When the screen showed a high reflective light that marked the cornea center, the subject was instructed to blink, and the image was acquired immediately after the image stabilized. Scan continuously for 3 times, measure the lower TMH with its own software caliper tool and take its average value. After the TMH was measured, the central thickness of the cornea was measured with the built-in OCT program. During the examination, the patient was instructed to look at the red light in front and the corneal light band was adjusted between the target band. The scan line length was set to 3 mm and the horizontal scan was performed and the image was captured and saved. The patient did not use eye drops within 2 h before the examination to exclude the effect of eye drops on the measurement of TMH.
- (6) Collection and determination of CXCR3 and CCR5: 10ul of the patient's tears were absorbed by a disposable capillary tear collector and placed in a 0.5 mL EP tube, stored at -80℃. The protein expressions of CXCR3 and CCR5 were detected by Elisa using Human CXCR3 ELISA Kit (ml027884, Mlbio, China) and Human CCR5 ELISA Kit (ml027980, Mlbio, China).

### Statistical analysis

The SPSS version 22.0 software was used for statistical analysis. Biologic parameter data of each group were compared pairwise by independent sample T test, and the normality test was performed by Kolmogorov–Smirnov test. One-way ANOVA was used to compare the Elisa gray values between the two groups, and the Levene test was used for the homogeneity of variance test. Pearson correlation analysis was used to test the correlation between biological parameters and the expression of CXCR3 and CCR5. All statistical results are expressed as mean ± standard error of the mean (SEM) and a *p* value < 0.05 was considered statistically significant.

## Results

### Comparison of visual acuity, IOP, CFS, BUT and Schirmer I test value at 1 day, 1 month and 3 months after injury in each group

As shown in Table 1, Visual acuity, IOP, CFS, BUT and Schirmer I tests of patients were performed on patients in the observation group at 1 day, 1 month and 3 months after injury, respectively. The same tests were performed in the control group on the first day of enrollment.

**Table 1:** Comparison of biological parameters at different times in each group

Group Parameter		Visual acuity (D)	IOP (mmHg)	CFS	BUT (s)	Schirmer I test (mm)
Group I (Grade I)	1 day	0.70 ± 0.06^*^	18.38 ± 1.00^*^	3.75 ± 0.53^*^	13.50 ± 0.87	13.75 ± 0.98
	1 month	0.85 ± 0.05	16.63 ± 0.65	0.88 ± 0.30^*#^	10.00 ± 0.76^#^	8.50 ± 0.42^#^
	3 month	0.95 ± 0.03^#^	16.50 ± 0.68	0.63 ± 0.18^*#^	8.75 ± 0.70^*#^	9.25 ± 0.37^*#^
Group II (Grade II)	1 day	0.58 ± 0.03^*^	19.25 ± 0.88^*^	8.25 ± 0.49^*^	15.00 ± 0.76	15.75 ± 1.00^*^
	1 month	0.80 ± 0.05	16.50 ± 0.82	1.13 ± 0.30^*#^	10.00 ± 0.27^#^	10.63 ± 0.53^#^
	3 month	0.88 ± 0.05^#^	16.00 ± 0.82^#^	0.75 ± 0.25^*#^	8.75 ± 0.65^*#^	8.88 ± 0.61^*#^
Group III (Grade III)	1 day	0.29 ± 0.03^*^	19.88 ± 0.58^*^	10.75 ± 0.49^*^	13.63 ± 0.57	18.88 ± 0.72^*^
	1 month	0.65 ± 0.07	16.13 ± 0.51	2.25 ± 0.31^*#^	8.50 ± 0.63^#^	11.13 ± 0.30^#^
	3 month	0.83 ± 0.06^#^	16.25 ± 0.37^#^	1.88 ± 0.23^*#^	6.88 ± 0.58^*#^	9.00 ± 0.38^*#^
Group IV (Grade IV- V)	1 day	0.06 ± 0.02^*^	22.33 ± 1.45^*^	12.00 ± 0.00^*^	14.00 ± 1.16	20.33 ± 0.88^*^
	1 month	0.10 ± 0.02^*^	17.67 ± 1.76	5.67 ± 0.88^*#^	10.67 ± 1.33^#^	11.67 ± 0.88^#^
	3 month	0.12 ± 0.01^*^	17.67 ± 0.88^#^	3.00 ± 0.58^*#^	6.67 ± 0.67^*#^	7.00 ± 0.58^*#^
Control group		0.93 ± 0.04	15.88 ± 0.52	0.00 ± 0.00	11.25 ± 0.73	12.38 ± 0.78

*Note*: a. Compared with the control group, ^*^p < 0.05; b. Compared with 1 day after injury in the same group, ^#^*p* < 0.05

#### Visual acuity

Compared with the control group, the visual acuity of patients with ocular acid burn in group I-IV significantly decreased 1 day after injury (all *p *< 0.05). 3 months after injury, there was no significant difference in visual acuity between patients in group I-III and those in the control group (all *p* > 0.05). And the visual acuity of patients in group IV was still significantly lower than those in the control group 3 months after injury (*p* < 0.05). Compared with 1 day after injury in the same group, the visual acuity of patients with ocular acid burn in group I-III was significantly improved 3 months after injury (all *p* < 0.05) whereas the visual acuity of patients in group IV didn’t significantly improve 3 months after injury(*p* > 0.05).

#### IOP

Compared with the control group, IOP of patients with ocular acid burn in group I-IV was significantly increased 1 day after injury (all *p* < 0.05). Although the IOP in group I-III increased, it was still within the normal range while the IOP in group IV exceeded the upper limit of normal value. There was no significant difference in IOP between I-IV group and control group 3 months after injury (all *p* > 0.05), indicating that IOP in the four groups returned to the daily level three months later. Compared with 1 day after the injury in the same group, IOP of patients with ocular acid burn in group II-IV decreased significantly 3 months after the injury (all *p* < 0.05). However, there was no significant decrease in IOP of patients in group I (*p* > 0.05), which may be due to the mild degree of injury and the difference in IOP between 3 months after recovery and 1 day after injury was too small to be statistically significant.

#### CFS

Compared with the control group, patients with ocular acid burn in groups I-IV had significantly higher CFS scores at 1 day, 1 and 3 months after injury (all *p* < 0.05). When compared with 1 day after injury in the same group, the CFS scores of patients with ocular acid burn in the I-IV group were significantly reduced at 1 and 3 months after injury (all *p* < 0.05). Moreover, CFS scores in the I-IV group were lower at 3 months after injury than at 1 month after injury (all *p* < 0.05).

#### BUT

Compared with the control group, patients with ocular acid burn in groups I-IV had significantly shorter BUT 3 months after injury (all *p* < 0.05). 1 day after injury, however, BUT in groups II and III was longer than that in the control group (both *p* < 0.05) while BUT in groups I and IV was not significantly different from that in the control group (both *p *> 0.05). These results suggest that there was no regular difference in BUT between group I-IV and the control group 1 day after acid burn injury, which may be due to the high degree of corneal edema 1 day after injury that affected the timing of tear film rupture. When compared with 1 day after injury in the same group, BUT was significantly shorter at 1 and 3 months after injury in the I-IV group (all *p* < 0.05).

#### Schirmer I test

Compared with the control group, the values of Schirmer I test values were significantly higher in patients with ocular acid burn in groups II-IV 1 day after injury; while the values of Schirmer I test significantly decreased in patients with ocular acid burn in groups I-IV 3 months after injury (all *p* < 0.05). When compared with 1 day after injury in the same group, Schirmer I test values of patients with ocular acid burn in the I-IV group were significantly shorter at 1 and 3 months after injury (all *p* < 0.05).

### Comparison of corneal thickness at 1 day, 1 month and 3 months after injury in each group

**Fig. 1:**
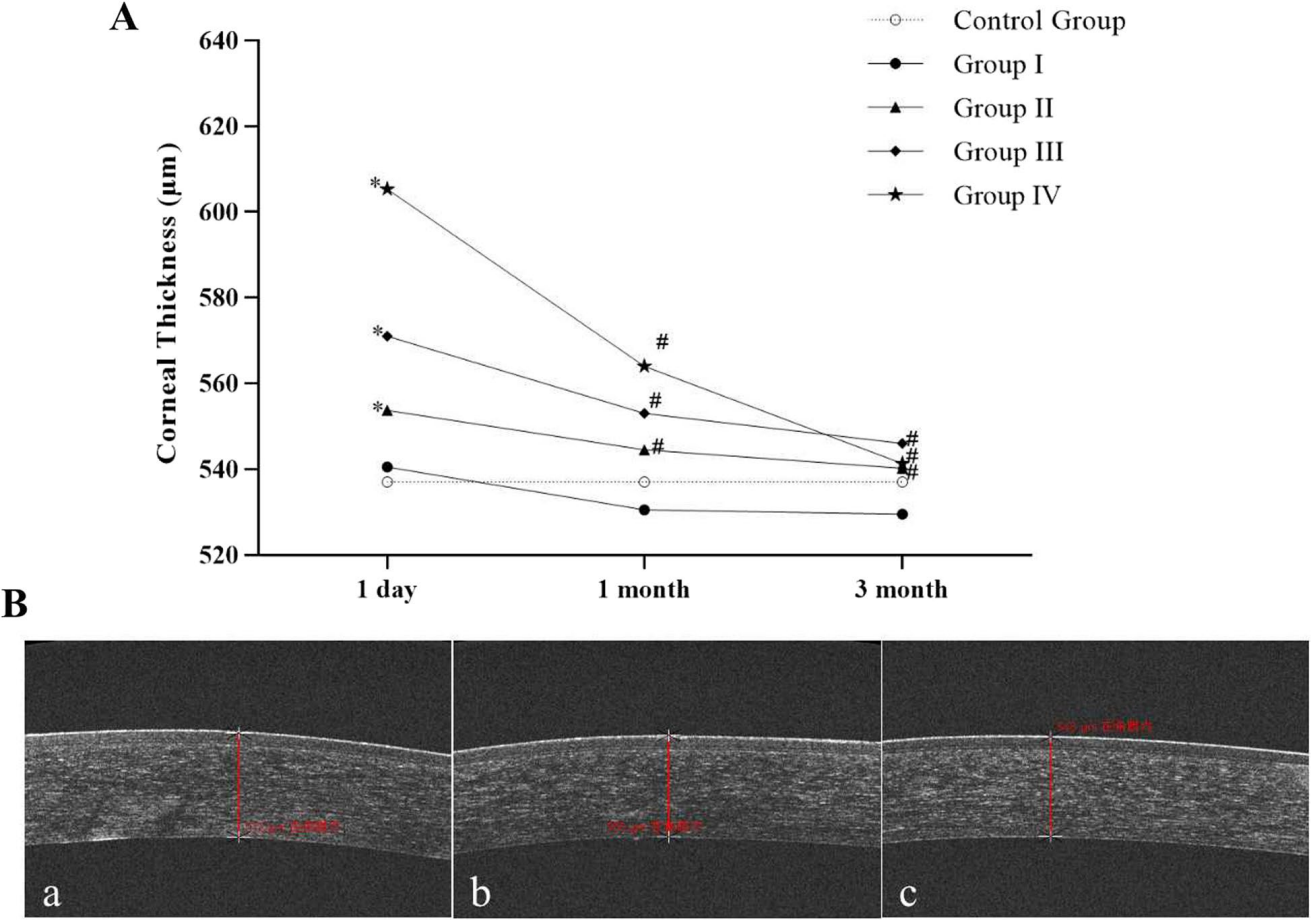
**A** Comparison of corneal thickness at 1 day, 1 month and 3 months after injury in each group. (Compared with the control group, ^*^p < 0.05; Compared with 1 day after injury in the same group, ^#^*p* < 0.05) **B** OCT image of corneal thickness of a patient with ocular acid burn in group III. (a. 1 day after injury; b. 1 month after injury; c. 3 months after injury.)

The comparison of corneal thickness at different time points in each group and OCT images of corneal thickness of a patient in group III are shown in Fig. 1. The corneal thickness of the control group was (537.00 ± 2.59) μm. 1 day after injury, the corneal thickness of patients with ocular acid burn in the I-IV group was (540.50 ± 4.50) μm, (553.75 ± 3.10) μm, (571.00 ± 3.36) μm, (605.33 ± 8.11) μm, respectively. The corneal thickness of patients with ocular acid burn in the II-IV group 1 day after injury was significantly thicker than that in the control group (all *p* < 0.05).

One month after injury, the corneal thickness of patients with ocular acid burn in the I-IV group were (530.50 ± 4.12) μm, (544.50 ± 2.44) μm, (553.00 ± 2.10) μm, (564.00 ± 4.62) μm, respectively. The corneal thickness of patients with ocular acid burn in II-IV group at 1 month after injury was significantly thinner than that at 1 day after injury (all *p* < 0.05).

Three months after injury, the corneal thickness of patients with ocular acid burn in the II-IV group were (529.50 ± 4.21) μm, (540.25 ± 2.52) μm, (546.00 ± 2.51) μm, (541.33 ± 3.53) μm, respectively. The corneal thickness of patients with ocular acid burn in II-IV group 3 months after injury was significantly thinner than that 1 day after injury (all *p* < 0.05).

### Comparison of TMH at 1 day, 1 month and 3 months after injury in each group

**Fig. 2:**
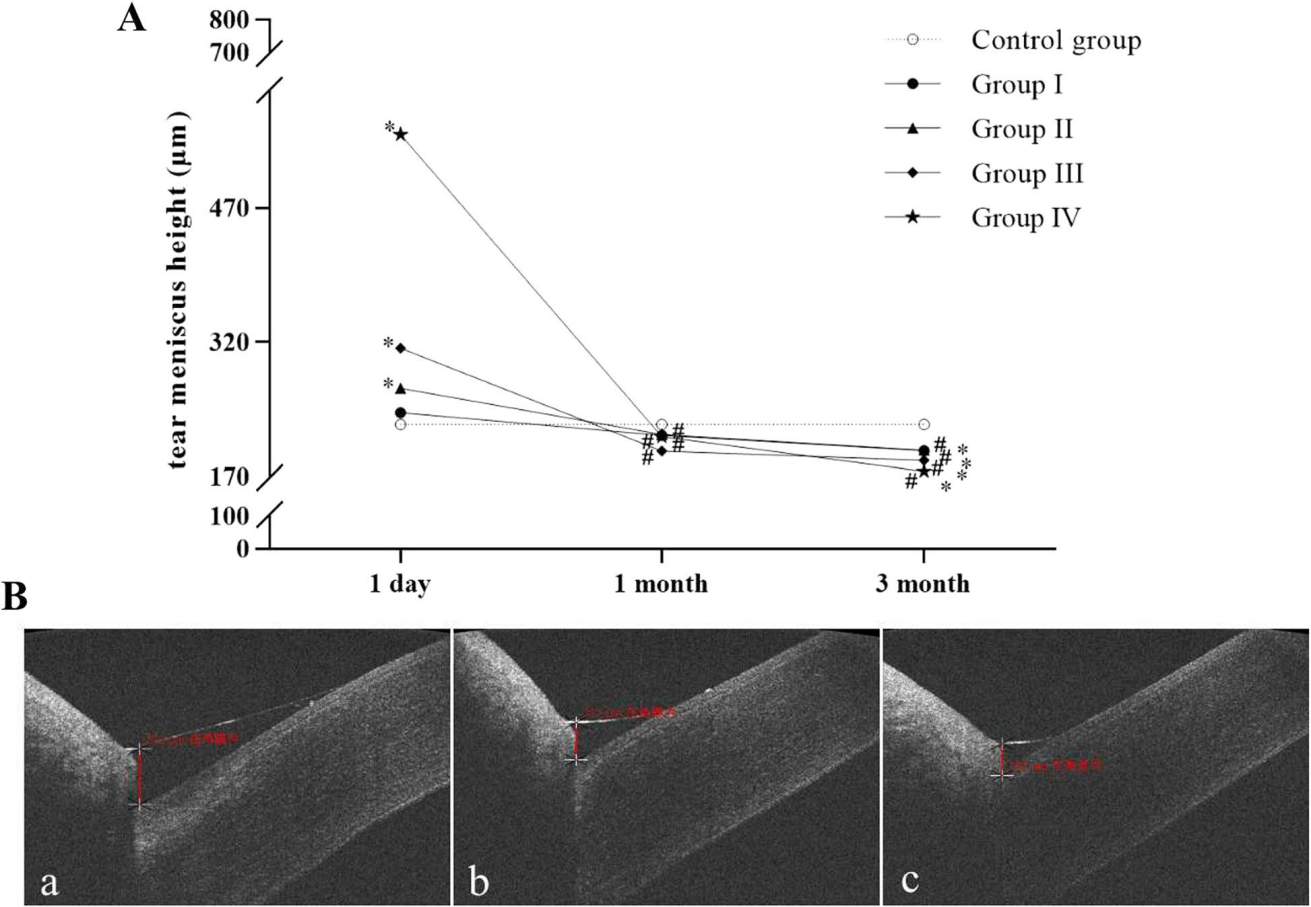
**A** Comparison of TMH at 1 day, 1 month and 3 months after injury in each group. (Compared with the control group, ^*^p < 0.05; Compared with 1 day after injury in the same group, ^#^*p *< 0.05) **B** OCT image of TMH of a patient with ocular acid burn in group III. (a. 1 day after injury; b. 1 month after injury; c. 3 months after injury.)

The comparison of TMH at different time points in each group and OCT images of TMH of a patient in group III are shown in Fig. 2. The TMH of the control group was 228.00 ± 6.93 μm. 1 day after injury, the TMH of patients with ocular acid burn in the I-IV group was 241.25 ± 7.70 μm, 268.50 ± 4.31 μm, 313.50 ± 11.90 μm, 552.00 ± 77.25 μm, respectively. 1 month after injury, the TMH of patients with ocular acid burn in the I-IV group were 216.00 ± 5.24 μm, 217.00 ± 6.67 μm, 198.50 ± 6.97 μm, 214.67 ± 16.38 μm, respectively. 3 months after injury, the TMH of patients with ocular acid burn in I-IV group were 199.00 ± 5.39 μm, 199.50 ± 8.80 μm, 188.50 ± 7.61 μm, 176.00 ± 6.11 μm, respectively. Compared with the control group, the TMH of patients with ocular acid burn in II-IV group significantly increased 1 day after injury (all *p* < 0.05), and patients with ocular acid burn in I-IV group had significantly lower TMH 3 months after injury (all *p* < 0.05). When comparing the TMH of patients with ocular acid burn in the same group, the TMH of patients in the I-IV group at 1 month after injury was significantly lower than that at 1 day after injury, and the TMH of patients in the I-IV group at 3 months after injury was significantly lower than that at 1 month after injury (all *p* < 0.05). (Fig. 2).

### Expression of CXCR3 and CCR5 of patients in each group at 1 day, 1 month and 3 months after injury

**Fig. 3:**
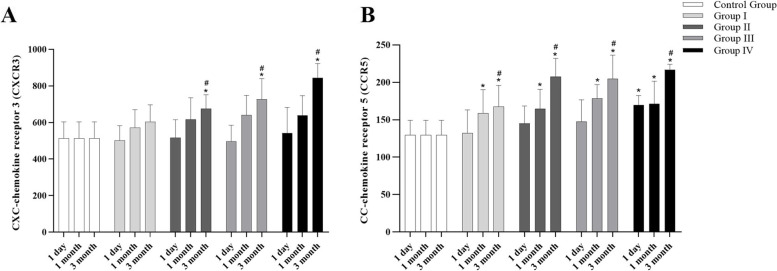
Comparison of CXCR3 and CCR5 protein expression in tears of patients with ocular acid burn in each group 1 day, 1 month and 3 months after injury. **A** Comparison of CXCR3 expressions in each group at a different time after injury. Compared with the control group, CXCR3 protein level in group II-IV significantly increased 3 months after injury (all *p* < 0.05). When compared with 1 day after injury in the same group, the protein levels of CXCR3 in the II-IV group significantly increased 3 months after injury (all *p* < 0.05). **B** Comparison of CCR5 expressions in each group at a different time after injury. Compared with the control group, CCR5 protein level in group I-IV significantly increased 1 month and 3 months after injury (all *p* < 0.05), and the CCR5 protein level in group IV was also significantly increased 1 day after injury (*p* < 0.05). When compared with 1 day after injury in the same group, the protein levels of CCR5 in the I-IV group significantly increased 3 months after injury (all *p* < 0.05)

The protein expression levels of CXC-chemokine receptor 3 (CXCR3) and CC-chemokine receptor 5 (CCR5) in tears of patients with ocular acid burn in each observation group were detected by ELISA at 1 day, 1 month and 3 months after injury. And the protein expression levels of CXCR3 and CCR5 in the tears of patients in the control group were detected after they were recruited. The results are as follows. (Fig. 3).

The protein levels of CXCR3 in the tears of patients in each group were compared after injury at different times. Compared with the control group, CXCR3 protein level in tears of patients with ocular acid burn in group II-IV was significantly increased 3 months after injury (all *p* < 0.05), and no significant change was found in the protein level of CXCR3 in tears of patients in group I 3 months after injury (*p* > 0.05). There was no significant difference in protein levels of CXCR3 in tears between the group I-IV and the control group 1 day and 1 month after injury (all *p* > 0.05). When compared with 1 day after injury in the same group, the protein levels of CXCR3 in tears of patients with ocular acid burn in group II-IV significantly increased 3 months after injury (all *p* < 0.05), but no significant change was observed in group I 3 months after injury (*p* > 0.05). And there was no significant difference in protein levels of CXCR3 of tears in group I-IV between 1 day and 1 month after injury (all *p* > 0.05). (Fig. 3A).

The protein levels of CCR5 in tears of patients in each group were compared at different times after injury. The protein levels of CCR5 in tears of patients with ocular acid burn in group I-IV was significantly higher than that in the control group at 1 month and 3 months after injury (all *p* < 0.05). The protein levels of CCR5 in tears of patients in group IV was significantly higher than that in the control group at 1 day after injury (*p* < 0.05). And there was no significant difference in the protein levels of CCR5 in tears between the group I-III and the control group 1 day after injury (all *p* > 0.05). When compared with 1 day after injury in the same group, the protein levels of CCR5 in tears of patients with ocular acid burn in the I-IV group significantly increased 3 months after injury (all *p* < 0.05), and no significant change was found in the protein level of CCR5 in tears of patients in group I-IV 1 month after injury (all *p* > 0.05). (Fig. 3B).

### Correlation analysis of CXCR3 and CCR5 protein expression levels with ocular biological parameters

**Fig. 4:**
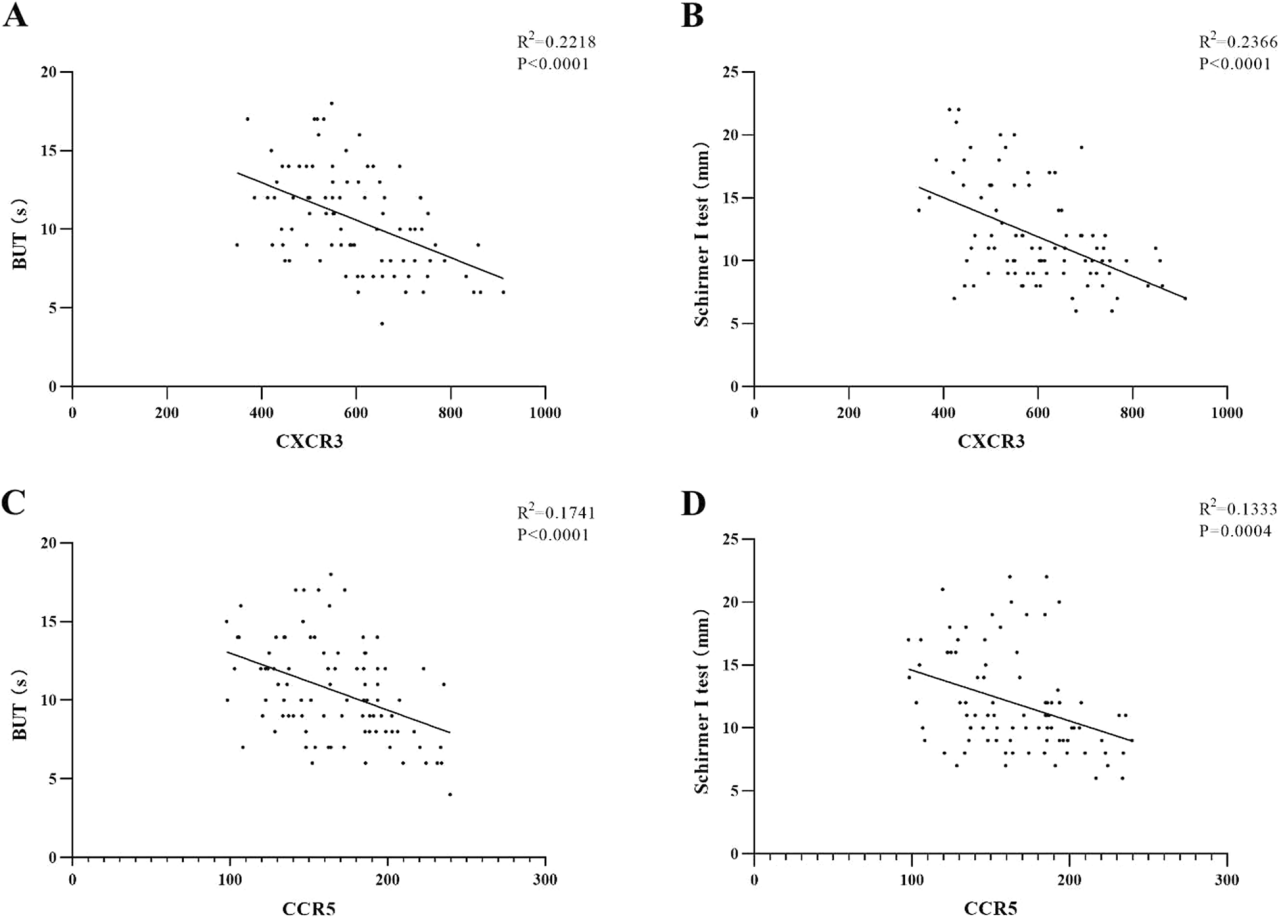
Correlation between CXCR3 and CCR5 protein expressions and biological parameters. The expression of CXCR3 was significantly negatively correlated with BUT (*R *= -0.4710, *P* < 0.0001), and CXCR3 was also significantly negatively correlated with Schirmer I test values(*R* = -0.4864, *P* < 0.0001). The expression of CCR5 was significantly negatively correlated with BUT (*R* = -0.4173, *P* < 0.0001), CCR5 was also significantly negatively correlated with Schirmer I test values(*R* = -0.3650, *P* = 0.0004)

The relationship between CXCR3 and CCR5 protein expressions in tears and ocular biological parameters of acid burn patients in each group at different time points after injury were analyzed by Pearson correlation. The GraphPad Prism 8 software was used to make correlation images. The expression of CXCR3 in tears of patients with ocular acid burn was significantly negatively correlated with BUT (*R* = -0.4710, *P* < 0.0001, Fig. 4 A), and the expression of CXCR3 in tears of patients with ocular acid burn was also significantly negatively correlated with values of Schirmer I test (*R* = -0.4864, *P* < 0.0001, Fig. 4 B). The expression of CCR5 in tears of patients with ocular acid burn was significantly negatively correlated with BUT (*R* = -0.4173, *P* < 0.0001, Fig. 4 C), the expression of CCR5 in tears of patients with ocular acid burn was also significantly negatively correlated with values of Schirmer I test (*R* = -0.3650, *P* = 0.0004, Fig. 4 D), and CXCR3 and CCR5 in tears of patients with ocular acid burn were not significantly correlated with other indicators.

## Discussion

The present study shows that ocular acid burn causes significant damage to the structure and function of the eye, resulting in significant changes in ocular biological parameters. Specifically, the visual acuity of patients with ocular acid burn decreased significantly 1 day after injury, and the visual acuity of patients with severe acid burn (grade IV burn and above) was irreversibly damaged after injury, and their visual acuity could not return to normal 3 months after injury. IOP increased one day after ocular acid burn, but returned to normal 3 months after injury. CFS scores increased significantly 1 day after ocular acid burn, but gradually decreased from 1 to 3 months after injury. BUT was significantly prolonged one day after ocular acid burn, but significantly shortened and lower than normal 3 months after injury. Similarly, Schirmern I test values increased significantly one day after ocular acid burn, but decreased significantly and were lower than normal 3 months after injury. Corneal thickness increased significantly 1 day after acid burn, but returned to normal 3 months after injury. TMH increased significantly one day after ocular acid burn, but decreased significantly and was lower than normal 3 months after ocular acid burn. In addition, the levels of CXCR3 and CCR5 in tears gradually increased to significantly higher than normal levels within 3 months of ocular acid burn. Notably, the values of BUT and Schirmer I test values increased significantly 1 day after injury, while CXCR3 and CCR5 levels in tears were almost normal. However, 3 months after the injury, CXCR3 and CCR5 were significantly increased, while BUT and Schirmer I test values were significantly decreased. And Pearson correlation analysis showed that CXCR3 and CCR5 expressions were significantly negatively correlated with BUT and Schirmer I test values. These results indicate that CXCR3 and CCR5 play an important role in the occurrence of dry eye after ocular acid burn.

Ocular acid burn is a chemical eye injury that can be divided into alkali burn and acid burn according to the etiology. Although acid injuries tend to be less severe than alkali injuries, it can still lead to a range of sequelae, including potential corneal ulceration/perforation, secondary open angle glaucoma, corneal scarring, limbal stem cell deficiency (LSCD), dry eye, etc. [10]. Studies have shown that chemical ocular injuries (acid and alkali burns) can cause extensive damage leading to visual impairment by causing widespread inflammation, scarring, melting, and necrosis of the ocular structures [11–13]. And severe periorbital edema or inflammation of the trabecular meshwork after a burn can lead to high intraocular pressure [11]. The inflammatory cascade after chemical ocular injury is mainly the activation of pro-inflammatory signaling pathway (NF-κB/ NLRp3-Caspase1-IL-1 β), followed by a series of downstream inflammatory events [14]. First, transcription factor—nuclear factor (NF-κB) and inflammatory body NLRP3 are increased, which activate macrophages and cause autoimmune responses. Subsequently, the increase of TNF-α, interleukin IL-1β, IL-10, chemokines CXCR3, CCR5 and other inflammatory cytokines mediates the aggregation of macrophages and neutrophils, resulting in inflammatory responses. At the same time, the decrease of fibroblast growth factor TGF-β makes the immune-suppression function and anti-inflammatory effects weakened. In our study, patients with various degrees of ocular acid burn had visual impairment, increased IOP, corneal edema and thickening, and corneal epithelium damaged 1 day after injury. And the more severe the acid burn, the more serious the impairment of visual acuity and IOP, the more serious the corneal edema and thickening, and the damage of corneal epithelium. In addition, dry eye is a common complication after chemical eye injury, and its mechanism still remains unclear [15]. It has been suggested that, on the one hand, the corneal epithelium damage after chemical burns leads to the lack of limbal stem cells, which destroys the normal ocular surface structure and weakens the tear secretion function [16]. On the other hand, due to the destruction of the meibomian gland in the tarsal conjunctiva, burn patients often lack lipid in tear and therefore are predisposed to develop evaporative dry eye [17]. And evaporative-type dry eye disease was found to have a close clinical correlation with tear cytokines and chemokines [18]. In addition, many studies have suggested that the mechanism of dry eye after chemical eye injury is related to the inflammation factors mediated inflammatory reaction, and blocking the inflammation factors mediated signaling pathway can reduce inflammation, thus decreasing the apoptosis of corneal epithelium in dry eye after chemical eye injury and improving wound healing [19–21]. Inflammatory factors involved in the immune inflammatory response of dry eye include cytokines, chemokines and soluble receptors, among which chemokines and their receptors are widely used in clinical and experimental studies of dry eye [22]. In this study, 3 months after injury, BUT of patients with ocular acid burn was significantly shortened, CFS scores significantly increased, the Schirmer I test value significantly reduced, and the TMH significantly reduced, indicating that ocular acid burn caused the long-term complication – dry eye. CFS scores reflect the condition of corneal epithelium damage, which is an important sign of LSCD [23]. This study showed that severe burn patients had higher CFS scores and more severe dry eyes 3 months after burn, suggesting that damage to corneal epithelium or LSCD is an important cause of dry eyes after acid burn. Notably, we detected the levels of CXCR3 and CCR5 in the tears of patients with ocular acid burns and analyzed their correlation with the biological parameters of the patients' eyeballs. We found a significant negative correlation between CXCR3 and CCR5 levels in tears of patients with ocular acid burns and values of BUT and Schirmer I tests. These results suggest that CXCR5 and CCR5 may be involved in the occurrence of dry eye after ocular acid burn.

CXCR3 and CCR5 are important classical chemokine receptors, which are cytokines that can cause immune inflammatory response by inducing lymphocyte migration through binding with ligands. Lymphocyte migration and phagocytosis are key mechanisms of immune inflammatory response, which is considered to be one of the important pathogenesis of dry eye [24], and CXCR3 and CCR5 play an important role in it [25, 26]. The nature and composition of tears play a critical role in the pathogenesis of dry eyes. It is urgent to study the expression and change of inflammatory factors in tears for the prevention and treatment of dry eye disease. Similar to the previous reports, in this study, chemokine receptors CCR5 and CXCR3 were found expressed in the tears of patients with dry eye [27]. However, how CCR5 and CXCR3 play a role in the development of dry eye after ocular chemical burn and whether the content changes of CXCR3 and CCR5 in tears related to the disease progression of dry eye requires further studies.

Choi’s study suggested that by blocking the chemokine receptors, CCR5 as well as CXCR3, and their specific ligands, it may be possible to modulate the manifestations of immunopathologic responses at the ocular surface in dry eye disease [28]. Li’s study reveals that topical application of infliximab can decline tear CXCR3 expression and improve the clinical and histological parameters [29]. Similarly, topical APN-derived short peptides (ADPs) could effectively decrease the production of inflammatory factors, such as CXCR3 and CCR5, and improve clinical signs of experimental eye dry or alkali burn [30]. Current evidence suggests that CXCR3 and CCR5 are potential therapeutic targets for dry eyes. Firstly, the extracellular ligand IL-6 directs T cell recruitment by regulating local chemokine secretion and chemokine receptor (CCR4, CCR5, CXCR3, etc.) expression on the CD3^+^ infiltrate, thus leading to the occurrence of dry eye [22]. Secondly, when the chemokine receptor CXCR3 (along with CCR5) expressed by Th1 cells binds to their specific ligands, such as INF-γ, they act as a central mediator to coordinate the localization of CD4^+^ T cells to the ocular surface to perform an immune response, causing dry eye occurrence [31]. Therefore, a reasonable assumption was that after ocular acid burn, the contents of inflammatory factors CXCR3 and CCR5 in the eyes of patients are increased, and their combination with ligand leads to recruitment of T1 lymphocytes on the ocular surface, which leads to immune inflammatory response on the ocular surface, thus forming the long-term complications of dry eye. Furthermore, when CXCR3 and CCR5 act as the virus cellular co-receptor, CXCR3 and CCR5 antagonists have been used in the treatment of HIV infection in clinical practice [32], indicating the modulation of the CXCR3 and CCR5 pathway might be a potential therapeutic strategy. In this study, CXCR3 and CCR5 in tears of patients with ocular acid burn were found to be closely related to the manifestations of dry eye, indicating that CXCR3 and CCR5 are involved in the occurrence of dry eye after ocular acid burn. Therefore, it deserves further exploration to reveal whether the regulation of CXCR3 and CCR5 pathways to control the immune inflammatory response can be used for the prevention and treatment of dry eye after ocular acid burn.

## Conclusion

This study found that ocular acid burns would not only damage visual acuity and intraocular pressure, but also lead to long-term complications of dry eyes. In addition, dry eye after ocular acid burn is closely related to the expression of chemokine receptors CXCR3 and CCR5 in tears. CXCR3 and CCR5 may be potential targets for the prevention and treatment of dry eye after ocular acid burn. However, further studies to investigate the mechanisms and functions of CXCR3 and CCR5 in dry eye after ocular acid burn are needed to explore better treatment.

## Data Availability

The datasets generated and/or analysed during the current study are not publicly available due to copyright and ethical issues but are available from the corresponding author on reasonable request.

## Abbreviations

CXCR3: CXC chemokine receptor-3
CCR5: CC chemokine receptor-5
CFS: Corneal fluorescein staining scores
IOP: Intraocular pressure
BUT: Breakup time of tear film
TMH: Tear meniscus height
ADPs: APN-derived short peptides
SEM: Standard error of the mean
